# Supplementary material for: Fibroblast growth factor receptor expression in hemangioblastomas: A novel therapeutic target
Source: PLoS One. 2025 May 20;20(5):e0323979. doi: 10.1371/journal.pone.0323979 (PMC12092013; doi:10.1371/journal.pone.0323979)
Supplement: S1 Table — (PDF) [file pone.0323979.s001.pdf]

S1 Table Primer sequences and annealing temperatures

Target	Forward Primer	Reverse Primer	Annealing Temperature (°C)
VHL exon 1a	AAGACTACGGAGGTCGAC	TTCTTCAGGGCCGTACTC	57
VHL exon 1b	AAGAGTACGGCCCTGAAG	CGATTGCAGAAGATGACCTG	57.7
VHL exon 1c	CCAGGTCATCTTCTGCAA	CTTCAGACCGTGCTATCG	57.7
VHL exon 2	CACCGGTGTGGCTCTTTAAC	TGGGCTTAATTTTTCAAGTGG	56
VHL exon 3	GCAAAGCCTCTTGTTCGTTC	CCATCAAAAGCTGAGATGAAA	56
