# Supplementary material for: Fibroblast growth factor receptor expression in hemangioblastomas: A novel therapeutic target
Source: PLoS One. 2025 May 20;20(5):e0323979. doi: 10.1371/journal.pone.0323979 (PMC12092013; doi:10.1371/journal.pone.0323979)
Supplement: S2 Table — (PDF) [file pone.0323979.s002.pdf]

S2 Table Mutation status of *VHL* gene

Nucleic acid change	Amino acid change	Number of mutations in series	Number of mutations in COSMIC
c.110A>T	p.Glu37Val	2[a]	0
c.187C>A	p.Leu63Met	1	0
c.189G>A	p.Leu63Leu	1	0
c.190C>A	p.Arg64Ser	1	2
c.208G>T	p.Glu70X	1	12
c.229T>A	p.Cys77Ser	1	1
c.257C>T	p.Pro86Leu	1	8
c.277G>C	p.Gly93Arg	1	2
c.292T>A	p.Tyr98Asn	1	2
c.293A>G	p.Tyr98Cys	1	1
c.332G>T	p.Ser111Ile	1	4
c.333C>T	p.Ser111Ser	1	2
c.334T>A	p.Tyr112Asn	1	0
c.337C>T	p.Arg113X	2[a]	11
c.340G>C	p.Gly114Arg	1	8
c.397A>C	p.Thr133Pro	1	1
c.458T>A	p.Leu153Gln	1	1
c.462A>T	p.Pro154Pro	1	1
c.470C>T	p.Thr157Ile	1	5
c.481C>T	p.Arg161X	4[b]	42
c.482G>A	p.Arg161Gln	1	5
c.486C>A	p.Cys162X	1	5
c.490C>T	p.Gln164X	1	11
c.499C>T	p.Arg167Trp	2[a]	14
c.499C>G	p.Arg167Gly	3[c]	4
c.515C>T	p.Pro172Leu	1	0
c.548C>T	p.Ser183Leu	1	1
c.555C>G	p.Tyr185X	1	2
c.598C>T	p.Arg200Trp	1	7

Footnotes:
a: the mutations were from 2 different patients
b: 3 different samples from 1 patient, one sample from another patient
c: 2 samples from one patient, one sample from another patient
