# Supplementary material for: Fibroblast growth factor receptor expression in hemangioblastomas: A novel therapeutic target
Source: PLoS One. 2025 May 20;20(5):e0323979. doi: 10.1371/journal.pone.0323979 (PMC12092013; doi:10.1371/journal.pone.0323979)
Supplement: S3 Table — (PDF) [file pone.0323979.s003.pdf]

S3 Table Confounding factors

	FGFR3					FGFR4				
	neg	pos	total	p	Phi coefficient	neg	pos	total	p	Phi coefficient
**FGFR2**										
low[a]	55	7	62	0.898[c]	0.011	27	34	61	0.379[c]	0.075
high[b]	66	9	75			28	48	76		
**FGFR3**										
neg	-	-				45	75	120	0.150[c]	minus 0.123
pos	-	-				9	7	16		

Footnotes:
a. Scores 0 or 1
b. Scores 2 or 3
c. Chi-square
