# Supplementary material for: Fibroblast growth factor receptor expression in hemangioblastomas: A novel therapeutic target
Source: PLoS One. 2025 May 20;20(5):e0323979. doi: 10.1371/journal.pone.0323979 (PMC12092013; doi:10.1371/journal.pone.0323979)
Supplement: S4 Table — (PDF) [file pone.0323979.s004.pdf]

S4 Table Association of VHL status with tumor characteristics

	Clinical VHL					VHL mutation				
	neg (n=84)	pos (n=33)	total	p		neg (n=57)	pos (n=28)	total	p	
**Sex**					Phi coefficient					Phi coefficient
male	54	17	71	0.901[a]	0.012	33	21	54	0.124[a]	minus 0.167
female	30	10	40			24	7	31		
**Clinical VHL**										Phi coefficient
neg	-	-				45	16	61	0.036[a]	0.228
pos	-	-				12	12	24		
**Location**					Cramer's V					Cramer's V
cerebellum	61	21	82	0.008[b]	0.310, df 3	40	18	58	0.096[b]	0.278, df 3
spinal cord	7	12	19			10	4	14		
cerebrum	5	1	6			3	1	4		
brain stem	8	7	15			2	6	8		
**Age at first diagnosis**					Effect size r					Effect size r
mean rank (range)	60.36 (24-78) (n=84)	42.44 (10-83) (n=27)		0.012[c]	0.238	46.36 (16-83) (n=57)	36.16 (16-73) (n=28)		0.073[c]	0.194
**Greatest diameter (mm)**					Effect size r					Effect size r
mean rank (range)	47.80 (5-50) (n=71)	42.12 (1-31) (n=21)		0.391[c]	0.089	36.02 (1-50) (n=49)	40.40 (9-48) (n=25)		0.406[c]	0.097
Not available						8	3			

Footnotes:
a. Chi-square
b. Fisher-Freeman-Halton Exact test
c. Mann-Whitney *U* test
df, degrees of freedom
