# Supplementary figures and images for: Fibroblast growth factor receptor expression in hemangioblastomas: A novel therapeutic target
Source: PLoS One. 2025 May 20;20(5):e0323979. doi: 10.1371/journal.pone.0323979 (PMC12092013; doi:10.1371/journal.pone.0323979)

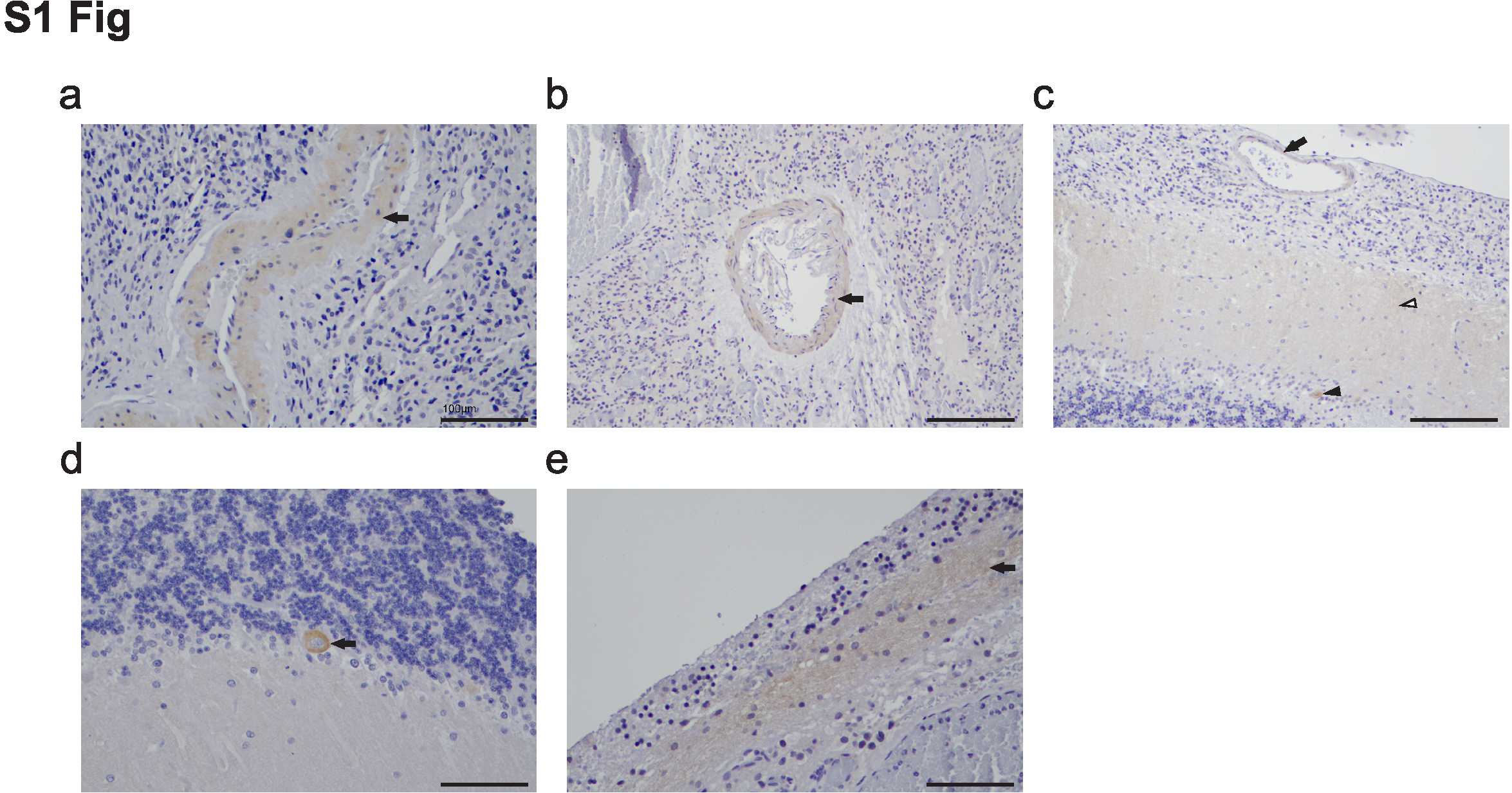

Supplement: S1 Fig — Smooth muscle of the blood vessels stained positive. Scale bar = 100 µm. c. The black arrowhead points to a positively stained Purkinje cell. The white arrow indicates an area where the white matter of the cerebellum is positively stained. The black arrow marks a blood vessel with positively stained smooth muscle. Scale bar = 100 µm. d. Purkinje cells were positively stained. Scale bar = 50 µm. e. The white matter of the cerebellum stained positive. Scale bar = 100 µm. (TIF) [file pone.0323979.s005.tif]

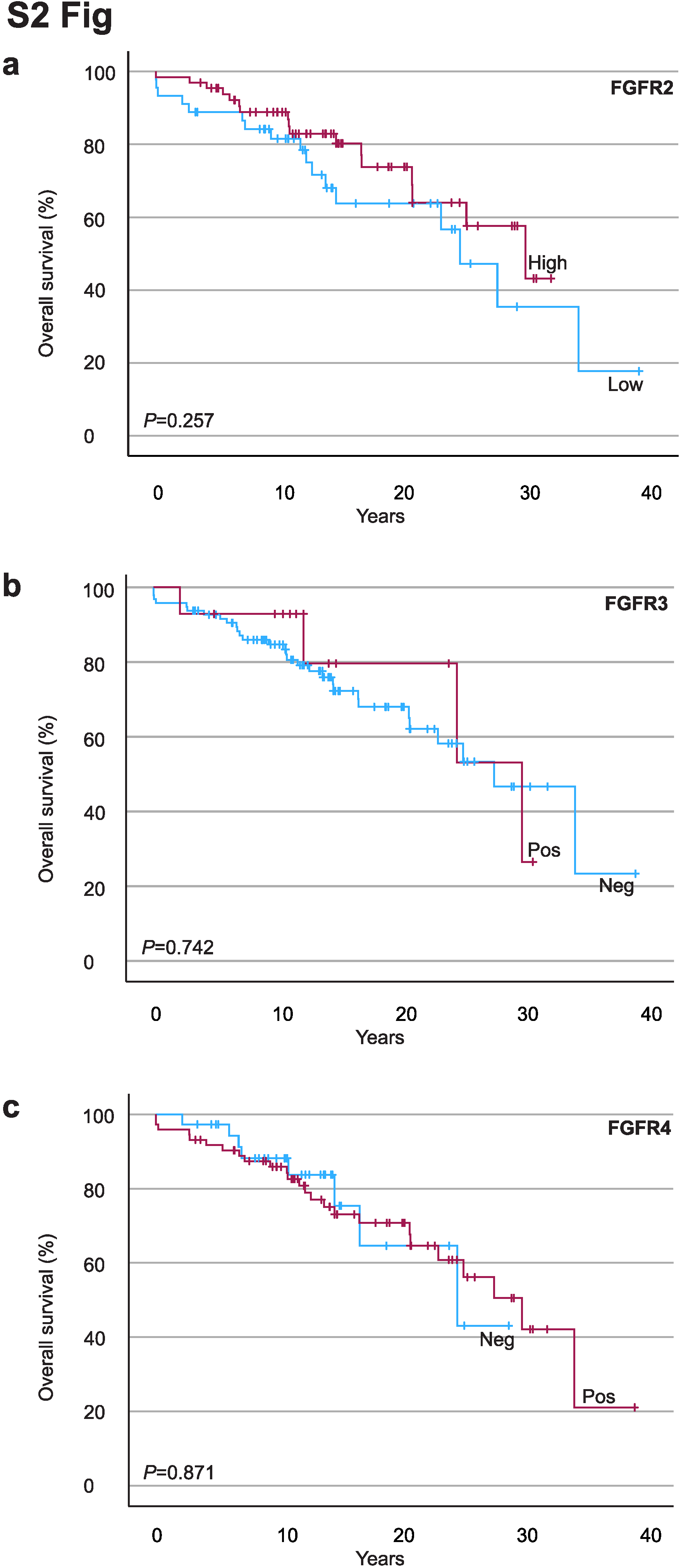

Supplement: S2 Fig — Association between FGFR2 expression and overall survival, b. association between FGFR3 expression and overall survival, c. association between FGFR4 expression and overall survival. (TIF) [file pone.0323979.s006.tif]
