# Supplementary material for: Fibroblast growth factor receptor expression in hemangioblastomas: A novel therapeutic target
Source: PLoS One. 2025 May 20;20(5):e0323979. doi: 10.1371/journal.pone.0323979 (PMC12092013; doi:10.1371/journal.pone.0323979)
Supplement: Attachment — Submitted filename: Reviewers Comments_PONE-D-24-35229.docx [file pone.0323979.s007.docx]

Dr. Md Shaifur Rahman

In the current study authors investigates the expression patterns and clinical relevance of fibroblast growth factor receptors (FGFRs) 1-4 in hemangioblastomas—benign but highly vascularized tumors of the central nervous system (CNS). The research aims to determine whether FGFRs, particularly FGFR2 and FGFR4, are differentially expressed in sporadic vs. von Hippel–Lindau (VHL)-associated hemangioblastomas and whether these receptors might serve as therapeutic targets. The study utilizes immunohistochemistry and molecular analysis on a large set of 139 tumor samples to assess receptor expression, investigate correlations with clinical factors (e.g., sex, tumor size, VHL mutations), and explore their potential role in precision medicine for hemangioblastoma treatment. Overall, the paper suggests that FGFRs may play a role in the development and progression of hemangioblastomas, and that further research is needed to fully understand their role and to explore their potential as therapeutic targets. The manuscript titled "Fibroblast growth factor receptors 2 and 4 are frequently expressed in hemangioblastoma" provides a well-conducted study on FGFR expression in hemangioblastoma. While the topic is relevant and interesting, the manuscript could benefit from improvements in language, clarity, scientific rigor, and structure. Below are specific observations. The following comments should be also considered during revision of the manuscript. I believed the manuscript should not be published in its current form and needs some clarification:

PLOS ONE criteria:

► Originality: The study presents original research, but the authors could strengthen their case by providing a clearer explanation of how their study builds upon existing knowledge in the field.

► Novelty: The results reported have not been published elsewhere, but the authors could consider discussing how their findings relate to existing literature on the topic.

► Technical standard: The experiments, statistics, and other analyses are not described in sufficient detail, making it difficult to assess their technical standard.

► Conclusions: The conclusions are not clearly supported by the data, and the authors could consider providing more detailed explanations of how their results relate to their conclusions.

► Intelligibility: The article is not presented in an entirely intelligible fashion, with some sections being unclear or difficult to follow.

Comments and recommendations:

► The manuscript could benefit from a thorough proofread to correct minor errors in grammar, punctuation, and formatting. For example, there are several instances of missing articles, incorrect verb tenses, and inconsistent formatting of headings and subheadings.

► The study addresses an important gap related to FGFRs in hemangioblastoma. However, the research gap and objectives are not explicitly highlighted in the introduction.

► The introduction could be strengthened by providing a clearer background on the significance of hemangioblastomas and the role of FGFRs in tumor development.

► Expand on the biological significance of FGFRs in tumor growth and angiogenesis resistance to VEGF therapies.

► The FGFR1-negative result should be discussed in relation to other studies. Are there other tumors where FGFR1 is similarly inactive?

► Some sections are slightly verbose, especially in the abstract and discussion. Reducing redundancy will enhance clarity.

► Some of the statistical conclusions require further elaboration to strengthen their relevance.

► The conclusion could be improved by summarizing the main findings and implications of the study. Consider adding a final thought on the significance of the study and its contributions to the field.

**Title**:

► Comment: The title is clear and concise but could be improved by adding subtitles to provide more context. For example, "Fibroblast Growth Factor Receptors (FGFRs) Expression in Hemangioblastomas: A Novel Therapeutic Target"

**Abstract:**

**Line 1:** “Hemangioblastoma is a highly vascularized benign tumor...”

- **Comment:** The description is concise but needs more specificity. Mention the risks associated with the tumor, like hemorrhage and recurrence, which create a clinical need for alternative treatments.

**Line 3:** “...complete removal is not always feasible, necessitating alternative strategies.”

- **Comment:** Avoid vague phrasing like “alternative strategies.”. Specify what strategies you are referring to (e.g., targeted therapies, FGFR inhibition). Instead: “...necessitating exploration of FGFR-targeted therapies or angiogenesis inhibitors.”

**Line 9:**
"A significant increase in FGFR2 expression level was observed in tumors harboring a VHL mutation..."

- **Scientific Suggestion:** You could discuss the biological mechanisms through which FGFR2 might interact with VHL mutation pathways. Expanding on the molecular links between the FGFR and hypoxia-inducible factor pathways would deepen the relevance of the finding.

**Line 9:**
"A significant increase in FGFR2 expression level was observed in tumors harboring a VHL mutation..."

- **Comment:** Consider rephrasing to avoid redundancy: "FGFR2 expression significantly increased in VHL-mutated tumors."

**Line 13:**
"This finding highlights the potential of FGFRs as promising therapeutic targets..."

- **Comment:** Use a more assertive tone. Replace "highlights" with "supports" or "demonstrates" for a stronger conclusion.

• **Grammar:** “The distribution and significance of FGFRs in hemangioblastoma **is** yet to be investigated” → Should be **“are yet to be investigated”** (subject-verb agreement).

• **Scientific Improvement:** The statistical significance for FGFR2 in tumors with VHL mutations (p=0.034) is mentioned. Consider including confidence intervals to enhance clarity.

**Introduction:**

**Comment:** The introduction is not clearly structured and does not provide a clear background on the significance of hemangioblastomas and the role of FGFRs in tumor development. Consider adding more context to the introduction to explain the importance of studying FGFRs in hemangioblastomas.

► Mention drugs like **infigratinib** or **futibatinib** to connect findings with clinical therapies and show the relevance of your work.

► Provide a detailed explanation of the biological significance of FGFRs in VHL-mutated hemangioblastomas, which bypass VEGF pathways and explain resistance to anti-VEGF therapies.

► Clarify variability in VHL mutation frequency and cite relevant studies.

**Line 19:**
"The most common location is the cerebellum, and other locations include the brainstem, spinal cord, cerebrum, retina, and peripheral nerves."

- **Comment:** Consider separating peripheral and central nervous system structures for clarity: "Other central nervous system locations include the brainstem, spinal cord, and cerebrum, while peripheral involvement includes the retina and nerves."

**Line 30:**
"...has gained prominence as a target in personalized cancer therapy."

- **Comment:** Mention specific FGFR inhibitors (e.g., infigratinib, futibatinib) used in clinical trials for other cancers. This shows the translational potential of FGFR inhibition and can be linked to future therapeutic options for hemangioblastomas.

**Line 30:**
"...has gained prominence as a target in personalized cancer therapy."

- **Comment:** This is a strong claim. Consider adding more references to support the statement.

**Line 45:**
"They also knocked down HIF-α in VHL loss-of-function endothelial cells, which did not impede their angiogenic activity."

- **Scientific Comment:** This is a crucial point but needs further explanation. Why does HIF-independent angiogenesis driven by FGFR matter in the context of VHL-mutated hemangioblastomas? Consider elaborating on why this could explain resistance to VEGF-targeted therapies.

**Methods:**

**Line 90:** “FFPE samples were cut into 4 µm sections...”

- **Scientific Comment:** Specify how variability in sample preparation (e.g., staining intensity) was managed to ensure reproducibility. Was scoring done by multiple pathologists?

**Line 153:**
"Two samples were excluded from FGFR1 scoring due to occasional strong staining background..."

- **Comment:** Background staining can be a technical issue. Was there an attempt to optimize antibody dilution or perform antigen retrieval differently to improve FGFR1 staining? Addressing this could suggest better reproducibility.

**Line 180:**
"...samples were categorized into the following groups: FGFR2 low (score 0 or 1) or high (score 2 or 3)."

- **Comment:** It's important to justify these cutoffs based on existing literature or statistical analysis. Were these categories pre-defined based on prior studies, or was there an exploratory analysis? Justifying this strengthens the validity of the results.

**Line 188:**
"Male patients were more likely to exhibit FGFR4 expression (p = 0.02)."

- **Comment:** Was the impact of hormonal differences between male and female patients explored? FGFR expression can be regulated by hormone levels, so this could be an interesting angle to pursue in the discussion or future studies.

**Survival Analysis:**

- While mortality is rare in hemangioblastoma, survival analyses could be enhanced by exploring predictors like tumor location or resection completeness.

**Results**

**Line 193:**
"Tumors of the cerebrum showed a higher likelihood of positive staining for FGFR4 (p = 0.009)."

- **Comment:** Consider providing effect sizes or odds ratios alongside p-values for more informative statistical reporting.

**Line 193:**
"Tumors of the cerebrum showed a higher likelihood of positive staining for FGFR4 (p = 0.009)."

- **Comment:** Explore why cerebrum-localized tumors might have higher FGFR4 expression. Could it be linked to tumor microenvironment differences, such as blood-brain barrier permeability or vascular density?

**Line 223:**
"Approximately half of the samples displayed positive staining for FGFR4."

- **Comment:** This could be discussed in the context of FGFR4-related tumor biology in other cancers. Is there a known role for FGFR4 in tumor progression or angiogenesis that could support this finding? Connecting this to broader cancer biology would elevate the discussion.

**Discussion:**

**Comment**: The discussion could be strengthened by providing a more detailed analysis of the results and their implications for the field. Consider discussing the limitations of the study and potential avenues for future research.

► The discussion section lacks specific details, such as the potential mechanisms by which FGFRs may contribute to the development and progression of hemangioblastomas.

► Throughout the results, p-values are provided, but without context for the effect size or clinical significance. Strengthening statistical sections by providing effect sizes or confidence intervals will improve the interpretation.

► While FGFR2 and FGFR4 are reported as overexpressed, there is limited mechanistic discussion. Suggest exploring downstream pathways activated by FGFRs, which could contribute to hemangioblastoma growth or treatment resistance. Expand on FGFR signaling pathways: Integrate more mechanistic insights by discussing the MAPK and PI3K pathways that FGFRs activate and their role in tumor progression.

► Cite any studies that report sex-dependent FGFR expression in other tumors to strengthen your argument.

► Suggest future research on genomic and transcriptomic profiling of FGFRs to explore their therapeutic potential.

► Go beyond descriptive reporting of FGFR2 and FGFR4 expression—discuss the implications for tumor growth, angiogenesis, or potential drug resistance.

► Be more decisive in suggesting FGFR inhibitors for future clinical research, emphasizing the potential impact of your findings.

► Add a section highlighting any limitations (e.g., retrospective design, manual scoring bias) to pre-empt reviewer concerns and demonstrate scientific integrity.

**Line 220:** “FGFR2 was expressed in the majority of samples, while FGFR1 was negative.”

- **Scientific Comment:** This result should be discussed in relation to other tumors—does this pattern align with known FGFR expression in other CNS tumors?

**Line 240:**
"Together with this previous report, our result indicates a potential role of FGFR2 in the development and progression of VHL-mutated hemangioblastomas."

- **Comment:** It would be useful to mention specific signaling pathways involved (e.g., FGFR2-mediated MAPK or PI3K pathways). This would provide a more detailed mechanistic insight.

**Line 250:**
"Interestingly, in our cohort, male patients were more likely to exhibit FGFR2 and FGFR4 expression."

- **Comment:** This observation could be strengthened by citing any preclinical or clinical evidence supporting differential FGFR expression based on sex in other tumors. If no such evidence exists, suggest this as a potential avenue for future research.

**Line 260:**
"...exhibited a higher likelihood of positive staining for FGFR4, suggesting that the expression characteristics of FGFR may vary depending on tumor location."

- **Comment:** It may be worth exploring whether tumor location in the brain affects its access to growth factors, leading to differential FGFR expression. Adding speculation here opens up new hypotheses for future studies.

**Conclusion**

- **Comment:** Instead of “highlighting” potential targets, use stronger phrasing:
  - Example: “Our findings **indicate** that FGFR2 and FGFR4 are promising therapeutic targets...”

► Suggest conducting **clinical trials** using FGFR inhibitors to validate their potential in treating hemangioblastomas.

►Add a brief paragraph acknowledging the **retrospective nature** and the subjectivity of manual scoring.

**Formatting and Data Presentation**

► Table 3 is somewhat hard to read due to misaligned columns. Clearer formatting will improve readability.

► Ensure all figures mentioned (e.g., Fig. 1 and Fig. 2) are adequately described in the text. Also, the legend for Figure 2 is too brief and lacks sufficient detail to guide readers. Expand the legend by describing each panel thoroughly—what specific staining intensities are shown, what the comparison groups represent, and how scoring was performed. This will ensure clarity and alignment with high technical reporting standards.

This manuscript addresses a relevant topic with significant clinical potential. However, several areas need improvement in language, scientific rigor, and presentation. With these refinements, the study can make a valuable contribution to the field, and it can meet the rigorous standards required for PLOS ONE publication.
